# Ficolin-2 Defends against Virulent *Mycobacteria Tuberculosis* Infection *In Vivo*, and Its Insufficiency Is Associated with Infection in Humans

**DOI:** 10.1371/journal.pone.0073859

**Published:** 2013-09-09

**Authors:** Fengling Luo, Xiaoming Sun, Yubin Wang, Qilong Wang, Yanhong Wu, Qin Pan, Chao Fang, Xiao-Lian Zhang

**Affiliations:** 1 State Key Laboratory of Virology, Department of Immunology, Hubei Province Key Laboratory of Allergy and Immunology, Wuhan University School of Medicine, Wuhan, P. R. China; 2 Department of Anesthesiology, Wuhan University Zhongnan Hospital, Wuhan, P. R. China; Karolinska Institutet, Sweden

## Abstract

Human ficolin-2 (ficolin-2/P35) is a lectin complement pathway activator that is present in normal human plasma and is associated with infectious diseases; however, little is known regarding the roles and mechanisms of ficolin-2 during *Mycobacterium tuberculosis* (Mtb) infection. Here, we describe our novel findings that the ficolin-2 serum levels of 107 pulmonary tuberculosis (TB) patients were much lower compared with 107 healthy controls. *In vitro* analysis showed that ficolin-2 bound to the virulent Mtb H37Rv strain much more strongly than to the non-virulent *M. bovis* BCG and *M. smegmatis*. Ficolin-2 bound to the surface glycolipid portion of H37Rv and blocked H37Rv infection in human lung A549 cells. Opsonophagocytosis was also promoted by ficolin-2. Importantly, we found that administration of exogenous ficolin-2 had a remarkable protective effect against virulent Mtb H37Rv infection in both C57BL/6J and BALB/c mice. Ficolin-A (a ficolin-2-like molecule in mouse) knockout mice exhibited increased susceptibility to H37Rv infection. We further demonstrated that ficolin-2 could defend against virulent Mtb H37Rv infection at least partially by activating JNK phosphorylation and stimulating the secretion of interferon (IFN)-γ, interleukin (IL)-17, IL-6, tumor necrosis factor (TNF)-α, and nitric oxide (NO) production by macrophages. Our data provide a new immunotherapeutic strategy against TB based on the innate immune molecule ficolin-2 and indicate that ficolin-2 insufficiency is associated with higher susceptibility to infection in humans.

## Introduction

TB remains a major global health problem. One-third of the world’s population is infected with *Mycobacterium tuberculosis* (Mtb), the etiologic agent of TB, which kills over 1.7 million people a year. The only tuberculosis vaccine currently available is an attenuated strain of *Mycobacterium bovis*, termed bacillus Calmette–Guérin (BCG), which has variable and limited efficacy in tuberculosis-endemic regions. BCG provides little protection against adult lung tuberculosis, and protection in adults varies from zero to 80%. In particular, the increasing prevalence of multidrug resistant (MDR) TB has greatly contributed to the increased difficulties in the control of TB. There has been an increased incidence of drug-resistant TB cases among human immunodeficiency virus (HIV)-infected patient populations [1,2]. Therefore, the development of new anti-TB molecules or drugs without cross-resistance to known antimycobacterial agents is urgently needed.

Innate immune molecules limit the early stages of infection and involve many different recognition and effector mechanisms, including the complement system [3–5]. Mannan-binding lectin (MBL) and ficolins are two types of complement lectins (carbohydrate-binding proteins). These lectins are capable of recognizing microbial carbohydrates and activating the lectin complement pathway via a mechanism similar to that of the classical pathway; however, MBLs and ficolins use MBL-associated serine proteases (MASP) rather than C1r and C1s [3,4].

Ficolins are oligomeric defense proteins assembled from collagen-like stalks and fibrinogen-like domains that are able to sense danger signals, such as pathogens or apoptotic cells. Three members of the human ficolin family have been identified and characterized: ficolin-2/P35 (L-ficolin) [5,6], ficolin-3 (H-ficolin) [7], and ficolin-1 (M-ficolin) [8]. Two types of ficolins, designated ficolin A and ficolin B [9], have been identified in mice. Human ficolin-2 and ficolin-3 and mouse ficolin A are expressed mainly in the liver and are present in the circulation as serum lectins that recognize GlcNAc. Although the function of ficolin B, which is expressed mainly in the bone marrow, is unknown, evidence suggests that ficolin-2, ficolin-1, ficolin A, and ficolin B are closely related, especially ficolin-2 and ficolin A (serum-type) [5,8–10].

Human ficolin-2 (L-ficolin), encoded by the FCN2 gene, was first cloned and described as a type of lectin with a similar structure and function to that of MBL [3,6] that plays an important role in innate immunity. Ficolin-2 (with a molecular weight of 35 kDa for a single chain) has a lectin-like activity for GlcNAc, lipopolysaccharides, 1,3-β-D glucan, lipoteichoic acid (LTA), and various acetylated compounds [8–11]. Its binding specificity differs from that of MBL [3] because ficolin-2 is primarily bound to GlcNAc, whereas MBL is primarily bound to mannose.

Both ficolins and MBL are characterized by the presence of collagen-like and carbohydrate-binding domains in a subunit, although their carbohydrate-binding moieties are quite different. A fibrinogen (FBG)-like domain is present in ficolins, and a carbohydrate recognition domain is present in MBL. The FBG domain of ficolin-2 forms a globular structure, similar to the carbohydrate recognition domains (CRD) of MBL, and binds to sugar structures. MBL and ficolins activate the complement pathway via the binding of their CRD or FBG domains to the surface carbohydrates of pathogens, thereby initiating the activation of the lectin pathway [12,13]. Ficolins and MBL also show opsonic activity via the binding of their collagen-like domain to macrophages.

Although ficolin-2 was reported to bind specifically to some clinically important microorganisms, act as an opsonin, and promote the elimination of microbes through the elicitation of phagocytosis or the activation of the lectin pathway of complement *in vitro* [13–17], little is known regarding the roles and mechanisms of ficolins during Mtb infection either *in vitro* or *in vivo*. It is therefore necessary to further elucidate the immune roles and mechanisms of ficolin-2 in response to this important bacterium.

## Materials and Methods

### Patients, animals and samples

Written informed consents were approved by each participant. The study and informed consents were reviewed and approved by medical ethics committee of Wuhan University School of Medicine. The animal experimental protocols were performed in compliance with all guidelines and approved by the Institutional Animal Care and Use Committee of Wuhan University. Mice were housed in a temperature-controlled room. Fresh water and rodent diet were available at all times. All administrations and inoculations were performed under isoflurane anesthesia, and all efforts were made to minimize suffering. For cell or tissue harvesting, animals were sacrificed by overdose of isoflurane. In the survival rate study, animals showing terminal signs of plague which is characterized by hunched posture, reluctance to move and to respond to external stimuli were sacrificed by overdose of isoflurane. At the end of the study, animals were euthanized with an overdose of isoflurane.

A total of 107 pulmonary TB patients and 107 healthy donors were studied. The sera of patients with active pulmonary TB were obtained from the TB hospital of Wuhan and Wuhan Medical treatment center, China from 2008 to 2010. All subjects were unrelated Chinese of Han ethnicity. The average ages were 46.7 years for patients and 42 years for healthy donors. The gender ratios (male/female) were 68% for patients and 59% for healthy donors. All patients with signs and symptoms suggestive of TB had radiologic and bacteriologic evaluations. Patients receiving immunosuppressive regimens or known to be infected with human immune deficiency virus, hepatitis B virus, hepatitis C virus, or hepatitis D virus were excluded. Samples of healthy donors were obtained from the medical examination center of Zhongnan hospital at Wuhan University, based on clinical and laboratory findings with no signs or symptoms of TB and negative results from TB-specific IFN-γ ELISPOT analysis.

### Bacterial strains, cells, and reagents

*M. tuberculosis*, H37Rv [strain ATCC 25618], *M. bovis* BCG [strain ATCC 35734], and *M. smegmatis* [strain ATCC 700084] were purchased from the Beijing Biological Product Institute [18]. The human lung adenocarcinoma epithelial cell line A549 (ATCC, Rockville, MD) was used as a model of human type II alveolar epithelial cells. A549 and the mouse colon carcinoma cell line CT26 [19] were cultured in RPMI-1640 medium containing 10% fetal bovine serum (FBS). Human Embryonic Kidney 293 (HEK293T) cell line and 293FT (fast-growing HEK293T) derived from the 293F Cell Line were cultured in Dulbecco’s Modified Eagle’s medium supplemented with 10% FBS. Mannosylated lipoarabinomannan (ManLAM) was purified according to a previous report [20]. FITC-labeled anti-F4/80 and PE-anti-IFN-γ antibodies, fixation buffer, and permeability buffer were purchased from eBioscience. The goat anti-mouse PE-IgG antibody was purchased from Santa Cruz Biotechnology. The anti-GST monoclonal antibody was purchased from EarthOx. Rhodamine B was purchased from Sigma. The ELISA kits (IFN-γ, IL-4, IL-12, IL-6, IL-17A, and TNF-α) were purchased from eBioscience. The monoclonal antibodies (mAbs) against phospho-c-Jun N-terminal kinase (p-JNK) (G-7) (sc-6254) and JNK were purchased from Santa Cruz Biotechnology. The anti-ficolin-2 mAb GN5 was purchased from Hycult Biotechnology. The rabbit anti-ficolin-2 polyclonal antibody was prepared as described in our previous publication [21].

### Animals

Eight-week-old female C57BL/6J and BALB/C mice were purchased from the Animal Center of Wuhan University, China. The animal experimental protocols were approved by the Institutional Animal Care and Use Committee of Wuhan University. The mice were housed in a temperature-controlled room. Fresh water and rodent diet were available at all times. All of the administrations and inoculations were performed under isoflurane anesthesia, and all efforts were made to minimize suffering. For cell or tissue harvesting, the animals were sacrificed by overdose of isoflurane.

In the survival rate study, animals showing terminal signs of plague, which is characterized by hunched posture, reluctance to move, and reluctance to respond to external stimuli, were sacrificed by overdose of isoflurane. At the end of the study, the animals were euthanized with an overdose of isoflurane.

### Preparation of ficolin A KO mice

A targeting construct was produced to disrupt the ficolin A gene (FCNA) of C57BL/6J mice by homologous recombination according to our previous report [19]. FCNA KO mice are maintained by backcrossing to C57BL/6J, and the FCNA KO mice used in this study belong to the 15th filial generation [17,19].

### Eukaryotic expression plasmid construction

Human full-length ficolin-2 cDNA (GenBank Acc. NM015837) was amplified and subcloned in-frame into the *Eco*RI and *Hin*dIII sites of the mammalian expression vector pcDNA3.1(-)/Myc-HisA (Invitrogen) to produce the plasmid pcDNA3.1-ficolin-2. Construction of the pVAX-1-ficolinA plasmid was described by Fujimori et al. [22]. The sequences of the constructs were confirmed by restriction enzyme digestion along with DNA sequence analysis. All of the DNA preparations were produced using endotoxin-free purification columns (Qiagen).

### Ficolin-2 lentivirus expression system

The ViralPower™ Lentiviral Expression System was obtained from Invitrogen. This system allows the creation of a replication-incompetent, HIV-1-based lentivirus that can efficiently transduce mammalian cell lines [23]. The cDNA of ficolin-2 was amplified by PCR from pcDNA3-based expression vectors and cloned into the pLenti-6 lentiviral expression plasmid using *Bam*HI and *Mlu*I restriction sites. The downstream anti-blasticidin gene allows selection of transduced cells. The structure of pLenti6-ficolin-2 was confirmed by sequencing.

To generate high-titer viral stocks, 293FT cells were co-transfected (LipofectAMINE™ 2000) with pLenti6-ficolin-2 (3 µg) and a mixture (9 µg) of three packaging plasmids (pLP1, pLP2, and pLP/VSVG), which supply the structural and replication proteins required for the production of the lentivirus *in trans* [23]. Approximately 72 h after the beginning of transfection, the supernatants were harvested, cleared by brief spin, and stored at -80 °C.

### Purification of recombinant proteins

The recombinant GST-ficolin-2, GST-ficolin A, and GST proteins were purified as described previously [19,21]. Human full-length ficolin-2 cDNA (GenBank Acc. NM015837) or ficolin A cDNA (GenBank Acc. NC000068) was amplified and subcloned in-frame into the *Eco*RI and *Hin*dIII sites of the prokaryotic expression vector pGEX-KG (Invitrogen) to produce the plasmid pGEX-KG-ficolin-2 or pGEX-KG-ficolinA, respectively. The purified GST-ficolin-2, GST-ficolinA, and GST proteins were further treated with endotoxin-removing resin (containing 50 µg/ml polymyxin B) and examined by SDS-PAGE and western blotting. The recombinant GST-ficolin-2 protein was identified by native PAGE.

### Measurement of serum ficolin-2 concentrations

The sandwich enzyme-linked immunosorbent assay (ELISA) method was used to measure concentrations of serum ficolin-2 as previously described [19,21]. Briefly, 96-well ELISA plates were coated with 100 µl of rabbit anti-ficolin-2 polyclonal antibody (1:100 dilution) [21]. After incubation at room temperature (RT) for one hour, the solution was removed, and the plates were rinsed. After washing three times with 0.2% Tween-20 in phosphate-buffered solution (PBS), 100 µl of each serum sample or 100 µl of different concentrations of recombinant ficolin-2 protein (serial dilutions from 0.625 µg to 20 µg) were added and incubated at 37°C for 2 h [19]. The plates were washed three times and blocked with 5% bovine serum albumin (BSA) overnight. Subsequently, mouse monoclonal anti-human ficolin-2 GN5 (1:1000 dilution) (HyCult Biotechnology b.v.) was added to each well and incubated at 37°C for 1 h. The plates were washed three times and incubated with 100 µl of horseradish peroxidase (HRP)-conjugated goat anti-mouse IgG (1:1000 dilution). Color development was achieved by adding 100 µl/well of tetramethylbenzidine (TMB) chromogen substrate (Sigma). The reaction was terminated by adding 100 µl of 0.5 M H_2_SO_4_, and the OD at 450 nm was measured using an ELISA reader. Ficolin-2 concentrations were determined using a ficolin-2 standard curve that was constructed using different concentrations of ficolin-2 recombinant protein. Data were obtained from at least three independent experiments. All of the statistical data shown represent the mean ± SEM.

### Bacterial binding assay and flow cytometry

A total of 1×10^8^ CFU of heat-inactivated (65°C for 30 min) Mtb H37Rv were incubated with 20 µg of GST-ficolin-2, GST protein, or GST-ficolin-2 protein plus ManLAM at room temperature for 2 h, and the bacteria were washed 3 times. Subsequently, anti-GST mAb (EarthOx) (1:100 dilution) in PBS was added and incubated at 37 °C for 1 h, and the bacteria were washed 3 times. The bacteria were then incubated with goat anti-mouse PE-IgG antibody (Santa Cruz Biotechnology) at 37 °C for 30 min. The stained bacteria were washed and examined using flow cytometry.

To examine the blocking effect of ficolin-2 or ficolin A on the infection of Mtb H37Rv in A549 cells, 4 µg of pcDNA3-ficolin-2 or pVAX-1-ficolin A was transfected into 1×10^6^ A549 cells using Lipofectamine 2000. After transfection for 48 h, the cells were incubated with 1×10^7^ of rhodamine B-labeled H37Rv (Rho-H37Rv) bacteria at 37 °C for 1 h. The mixtures were washed, and the cells were analyzed using flow cytometry.

### Biolayer interferometry

Binding analysis was performed by biolayer interferometry using an Octet Red system (ForteBio, Inc.). Biolayer interferometry is conceptually similar to surface plasmon resonance experiments in that a protein of interest is immobilized on a biosensor surface and subsequently exposed to potential binding partners in solution. The binding of analytes to the immobilized protein changes the optical properties of the biosensors, leading to a shift in the wavelength of light reflected off the binding surface. Biotinylated GST-ficolin-2 or GST was used at a concentration of 50 µg/mL in sample diluted (SD) buffer (1×PBS, 0.02% Tween-20, 100 µg/ml BSA), loaded onto streptavidin-coated biosensors, and incubated at 25–30 °C with 1×10^9^ CFU/ml of Mtb H37Rv, BCG, and *M. smegmatis* that were heat-inactivated. All of the binding traces and curves were used after dual deduction of GST binding and buffer binding for each type of bacteria.

### Complement C4 deposition assay

Lectin pathway activation was quantified using the C4 deposition assay, which was modified according to previous reports [21]. Briefly, 96 well microtiter plates were pretreated with 0.5 µg/ml polylysine (50 µl/well) at room temperature (RT) for 5 min and subsequently washed once with TBS. Bacteria (10^8^ CFU) were mixed with 100 µl of fresh sera from healthy human donors diluted 1:1 in TBS buffer (20 mM Tris-Cl, 1 M NaCl, 10 mM CaCl_2_, 0.05% Triton X-100, 0.1% BSA, pH 7.4) [21]. Then, different concentrations of purified recombinant GST-ficolin-2, GST-ficolin A, or GST protein were added, and the mixtures were incubated at 4°C overnight. The bacteria were then washed thoroughly, and 0.1 µg of purified human C4 protein (Diagnostic Biosystems) was added and incubated at 37°C for 1.5 h. The bacteria were washed again, and FITC-conjugated rabbit anti-human C4c (Diagnostic Biosystems) (1:100 dilution) was added and incubated at room temperature for 30 min. The absorbance values were measured at 485 nm with fluorescence spectrophotometer (PE Company).

### Analysis of opsonization

For the opsonophagocytosis analysis using flow cytometry [24], BALB/C mouse peritoneal macrophages (1×10^6^ cells/well) were seeded in 6-well plates with RPMI-1640 media. A total of 1×10^7^ CFU of rhodamine B-labeled Mtb H37Rv were incubated with 20 µg of GST, GST-ficolin-2, GST-ficolin-2 plus Cytochalasin B (CytB), a well-known disruptor of actin polymerization that block phagocytosis but not attachment [25] or CytB alone at 4°C for 30 min. The mixtures were then added to each well and incubated at 37 °C in a 5% CO_2_ atmosphere for 1 h. The cells were harvested and washed, and FITC-labeled F4/80 was added. The cells were then incubated at room temperature for 1 h, and double-positive cells were analyzed using flow cytometry.

### Purification of neutrophils, macrophages, CD4^+^ T cells, and CD8^+^ T cells

CD4^+^ and CD8^+^ T cells were purified from splenocytes using the BD^TM^ IMag Mouse CD4+ and CD8+ T Lymphocyte Enrichment Set-DM and the BD^TM^ IMagnet (BD Biosciences Pharmingen, USA) via negative selection. Murine neutrophils and macrophages were harvested from peritoneal exudates at 3 h and 4 days, respectively, after i.p. injection of liquid thioglycollate medium (BD) [18].

### Measurement of nitric oxide (NO)

Purified neutrophils and macrophages were cultured in a 96-well plate (1×10^4^ cells/well) and stimulated with different concentrations of GST, GST-ficolin-2, or GST-ficolinA protein for 6, 12, 24, or 48 h at 37°C, respectively. The supernatants (50 µl) were harvested and mixed with 50 µl of Griess reagent I and 50 µl of Griess reagent II [26]. The nitrite concentration was determined by spectrophotometry (560 nm) according to the protocol recommended by the manufacturer (Beyotime Biotech, Jiangsu, P. R. China). NO data are expressed as the mean ± SD (nitrite) in μM.

### In vitro cytokine ELISA

A total of 1×10^6^ CD4^+^ T cells, CD8^+^ T cells, neutrophils, or macrophages were incubated with medium containing 20 µg/ml of GST, GST-ficolin-2, or GST-ficolinA at 37°C for 48 h. The cells were collected and lysed, and the supernatants were collected for analysis of TNF-α, IFN-γ, IL-17A, IL-4, IL-12, and IL-6 levels using an ELISA kit (eBioscience, San Diego, CA, USA) according to the manufacturer’s instructions.

### Detection of phosphorylated JNK

Murine macrophages were adhered to the wells of a 96-well plate for 2 h at 37°C. The wells were then washed to remove the non-adherent cells, and the adherent macrophages were incubated with GST-ficolin-2 (20 µg/ml), GST protein (20 µg/ml), or LPS (10 µg/ml) for 15 min, 30 min, or 60 min at 37°C. The cells were subsequently collected and lysed. The expression levels of phosphorylated-JNK (p-JNK) (46/54 kDa) and total JNK were detected by western blot analysis using anti-p-JNK (G-7) (sc-6254) and anti-JNK, respectively, at dilutions of 1:1000–1:2000. Horseradish peroxidase (HRP)-conjugated goat anti-mouse IgG was used as the secondary antibody.

### Measurement of the mouse serum TNF-α and IL-17 levels in vivo by ELISA

Wild-type C57BL/6 mice or FCNA KO mice were administered 100 µl of LPS (100 µg/ml) per mouse via tail vein injection, and the blood sample (100 µl) was collected from the orbital vein of each mouse at 0, 3, 6 or 10 h. Subsequently, the serum TNF-α and IL-17 levels were examined by ELISA.

### Animal model of acute tuberculosis (TB)

Mtb H37Rv bacteria were maintained on Lowenstein-Jensen (L-J) medium and harvested during the log phase of growth. Bacilli were washed in 0.05% Tween-80 saline and ground to uniformity before use [18]. Bacterial culture and animal tests were performed in the Animal Biosafety Level 3 Laboratory (ABSL-III) of the Wuhan University School of Medicine. The murine bacterial challenge protocols were approved by the Institutional Animal Care and Use Committee of Wuhan University.

Female C57BL/6J or BALB/C mice (7–8 weeks old) were randomly divided into four groups, including the infected control group, the pCDNA3.1-ficolin-2-treated group, the empty pCDNA3.1 vector control group, and the streptomycin (SM) (Sigma)-treated group (seven successive injections of 200 µg SM once per day). Six mice were used in each group. Each mouse was intravenously challenged with virulent Mtb H37Rv (1×10^6^ CFU/mouse) [18] [Sweeney, 2011 #1164]. On the day of infection, 10 µg of pCDNA3.1-ficolin-2 or the empty pCDNA3.1 vector was administered via intramuscular electroporation with an Electric Square Porator (TERESA, Shanghai, P. R. China) [18,19,27]. Aethocaine (125 µl of a 20 µg/ml solution) and 10 µg of plasmid (in 100 µl of solution) were injected into each mouse at a ratio of 1:4. The quadriceps femoris was stimulated using an electrode to allow for simultaneous DNA injection and electrotransfer at the same site. The expression of ficolin-2 in the spleen and muscles was detected by western blot analysis, and the serum ficolin-2 concentration was analyzed in each treated mouse by ELISA on days 0, 4, 7, and 10. The survival status of each mouse was observed and analyzed daily.

The FCNA KO mice were divided into three groups, including the infected control group, the pCDNA3.1-ficolin-2-treated group, and the pVAX1-ficolin-A-treated group. Each mouse was intravenously challenged with virulent Mtb H37Rv (1×10^6^ CFU/mouse). On the day of infection, 10 µg of pcDNA3.1-ficolin-2 or pVAX1-ficolin-A was administered using the above methods. The survival status of each mouse was observed and analyzed.

For the lentivirus-ficolin-2 expression system, female BALB/C mice (7–8 weeks old) were randomly divided into three groups (n=6 per group), including the infected control group, the lentivirus-ficolin-2-treated group, and the lentivirus control group. Each mouse was intravenously challenged with virulent Mtb H37Rv (1×10^6^ CFU/each mouse). On the day of infection, 1×10^6^ transducing units (TU)/ml of lentivirus-ficolin-2 or lentivirus (mock) control were injected intravenously into each mouse. The survival time of each mouse was observed and analyzed daily.

### Bacterial enumeration in organ homogenates

Mice infected with Mtb H37Rv were sacrificed by overdose of isoflurane at 30 days post-infection, and the spleens and lungs were harvested and weighed. Tissues from individual mice (eight animals/group) were homogenized in saline (NS) (1 ml per 0.1 g of tissue). For every sample, 0.1 ml of the homogenate fraction was submitted to serial 10-fold dilutions in normal saline. Viable cells were enumerated.

### Histologic examination and acid-fast bacilli staining of lung tissue

After four weeks of Mtb H37Rv infection, the mice were sacrificed for histopathological analysis. Lung tissues were fixed with 4% (v/v) paraformaldehyde and embedded in paraffin for sectioning. The tissues were subjected to hematoxylin and eosin (H&E) or acid-fast bacilli staining and evaluated using light microscopy (400 × magnification).

### Statistical analysis

The data were analyzed with the SPSS software, and the Kaplan-Meier method was used to plot survival curves for each group. All data were tested for normal distribution and analyzed by Student’s *t* test and ANOVA, and *p*-values <0.05 were considered to be statistically significant.

## Results

### TB patients exhibit significantly lower serum ficolin-2 concentrations

**Figure 1:**
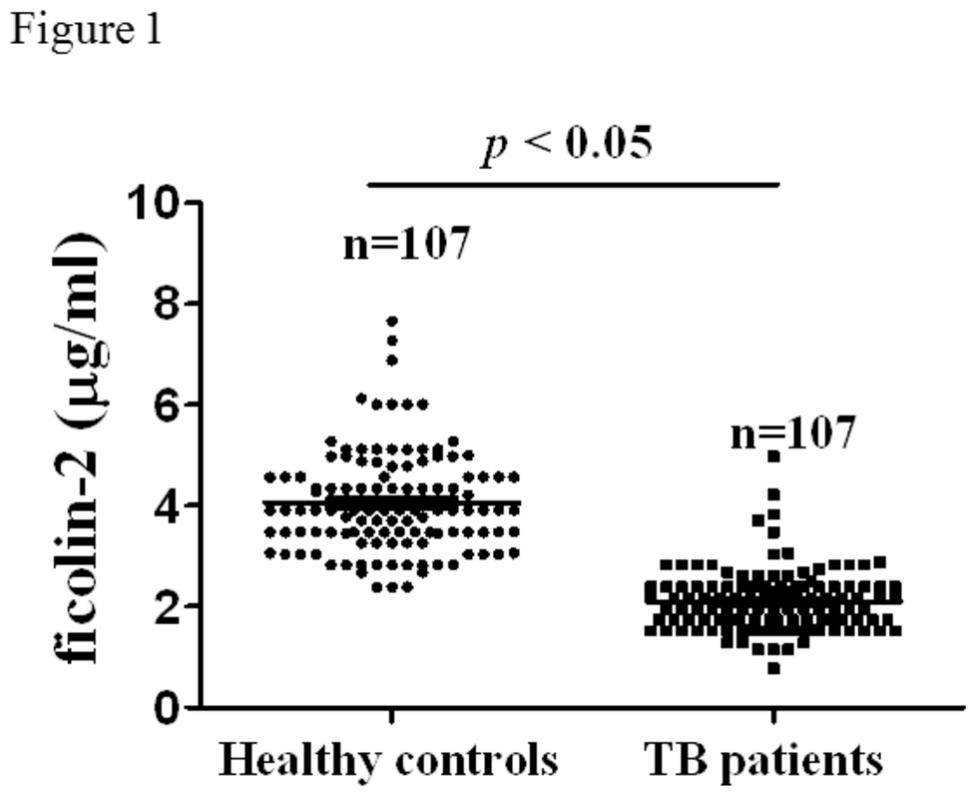
Comparisons of serum ficolin-2 levels in tuberculosis patients and healthy donors. Serum ficolin-2 concentrations in the TB patients (n=107) vs. those in the healthy donors (n=107), * *p* < 0.05. The data shown are the mean values of three experiments. All data were analyzed by one-way ANOVA with Bonferroni post-test.

Serum ficolin-2 concentrations were examined by a sandwich ELISA method using samples from 107 TB patients and 107 healthy donors. The serum level of ficolin-2 in TB patients was significantly lower than that in healthy donors (TB patients vs. healthy donors, *p* < 0.05, *t*-test) (Figure 1). The mean concentrations of ficolin-2 were 4.1 µg/ml and 2.07 µg/ml in healthy donors and TB patients, respectively. Our results regarding the ficolin-2 concentrations of healthy donors were similar to those of a previous report, which showed that ficolin-2 in the sera of healthy donors was 3.9–4.7 µg/ml [28,29]. These findings suggest that a ficolin-2-deficient host might be more susceptible to infection by Mtb H37Rv.

### Ficolin-2 recognizes and binds to the surface glycolipid of virulent Mtb H37Rv

**Figure 2:**
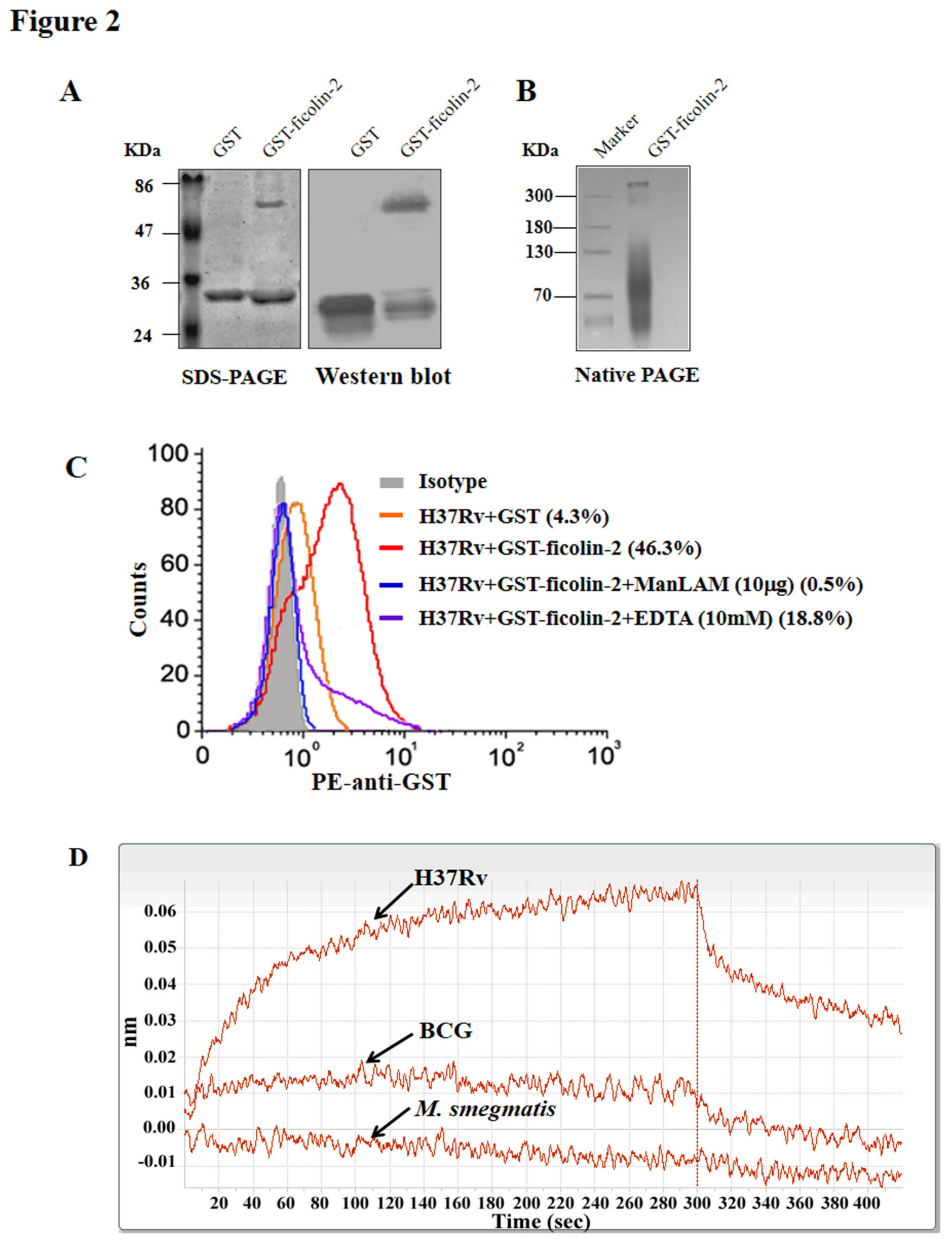
Ficolin-2 recognizes and binds to a surface glycolipid of the virulent Mtb H37Rv. (A) Analysis of the purified recombinant GST-ficolin-2 protein by SDS-PAGE and western blotting using anti-GST mAb. (B) Analysis of the recombinant GST-ficolin-2 protein by native PAGE. (C) Flow cytometry analysis of the binding abilities of GST-ficolin-2/GST (20 µg) with 1×10^8^ CFU Mtb H37Rv in the presence of 10 µg of ManLAM in TBS-Ca^2+^ buffer or in the presence of 10 mM EDTA. PE-anti-GST mAb was used. (D) Examination of the binding responses of different Mtb strains and ficolin-2 protein, in parallel, using Octet streptavidin (SA) sensors. Biotinylated GST-ficolin-2 (biotin-GST-ficolin-2) or biotin-GST proteins were loaded onto SA sensors. The SA sensors were incubated with 10^9^ CFU/mL of H37Rv, BCG, or *M. smegmatis* for 300 s. All of the binding traces and curves were used after dual deduction of GST binding and buffer binding for each type of bacteria. The experiments were repeated three times.

Recombinant GST-ficolin-2 and GST proteins were prepared and characterized by SDS-PAGE and western blot (Figure 2A). GST-ficolin-2 protein was also identified by native PAGE, and the results indicated that GST-ficolin-2 might form multimers with higher molecular weights (MWs) (> 300 kDa) (Figure 2B). Similar results were obtained for recombinant GST-ficolinA protein. We next attempted to determine whether recombinant ficolin-2 protein could bind to virulent Mtb H37Rv using flow cytometry. We observed that ficolin-2 bound to the Mtb H37Rv strain, whereas the control protein GST could not bind to the bacteria (Figure 2C), and their binding could be blocked by the purified glycolipid ManLAM of Mtb H37Rv (Figure 2C). These findings indicate that ficolin-2 may bind to the surface glycolipid portion of H37Rv. In addition, our data (Figure 2C) showed that binding was also inhibited by the addition of EDTA, a Ca^2+^ chelator, indicating that ficolin-2 is a Ca^2+^-dependent lectin.

The binding interaction between Mtb and ficolin-2 was also analyzed using biolayer interferometry as described in the Materials and Methods. The results showed that ficolin-2 had a much higher binding affinity for H37Rv compared with BCG and *M. smegmatis* (Figure 2D). Ficolin-2 had a weak binding affinity for BCG and nearly no binding interaction with *M. smegmatis* (Figure 2D).

### Ficolin-2 inhibits Mtb H37Rv infection in lung-derived A549 type II alveolar epithelial cells

**Figure 3:**
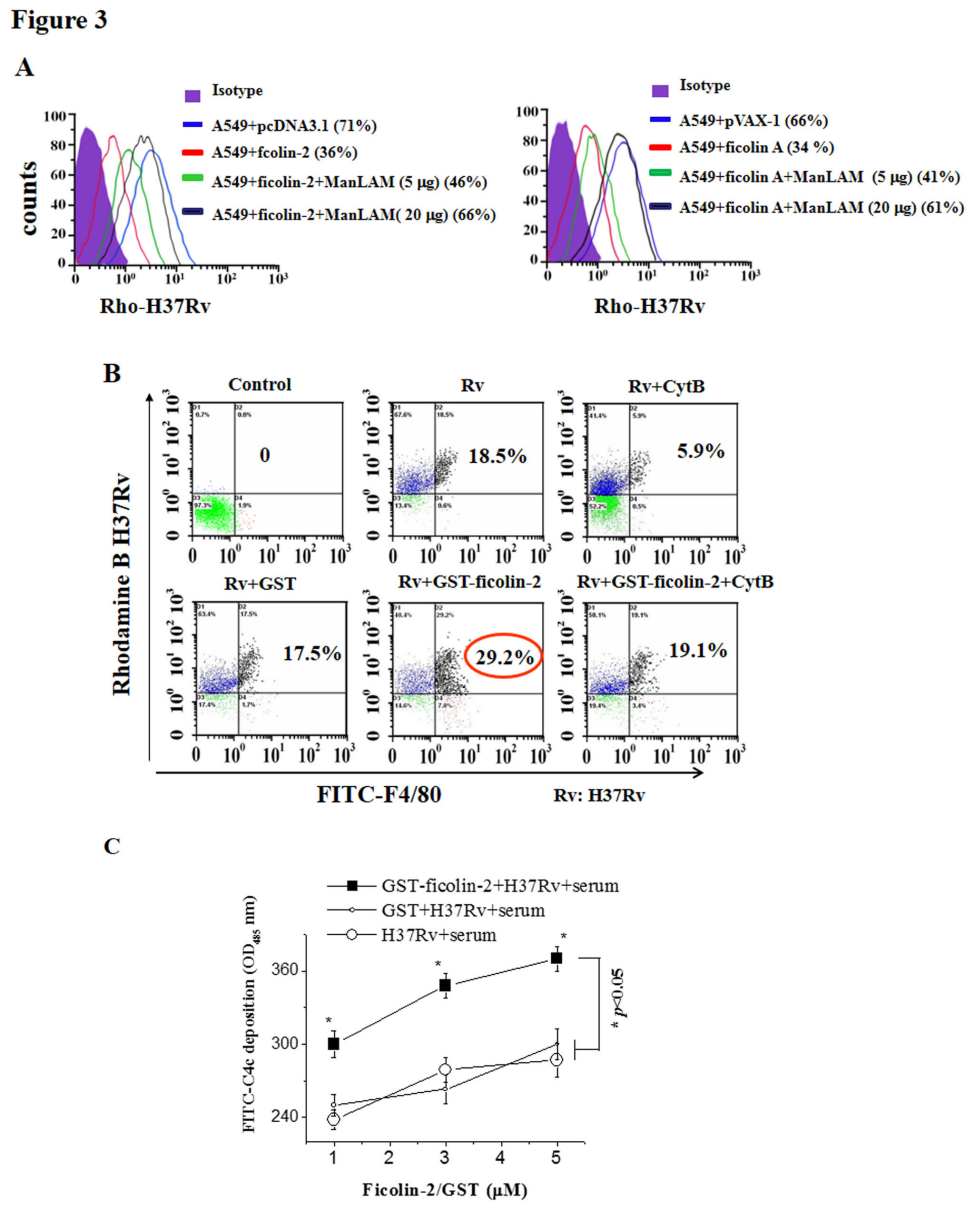
Ficolin-2 inhibits Mtb H37Rv infection and stimulates opsonization of macrophages. (A) Ficolin-2/ficolin A inhibited Mtb H37Rv infection of human lung A549 cells *in vitro*. pcDNA3.1-ficolin-2 or pVAX-1-ficolin-A plasmids were transfected into A549 cells for 48 h. Rhodamine B-labeled Mtb H37Rv (RhoB-H37Rv) was mixed with ficolin-2- or ficolin A-transfected human lung A549 cells with or without ManLAM, and the cells were analyzed using flow cytometry. (B) Ficolin-2 stimulated opsonization of macrophages. RhoB-H37Rv bacteria were incubated with 20 µg of GST, GST-ficolin-2, GST-ficolin-2 plus CytB, or CytB, respectively, at 4°C for 30 min. The uptake of the H37Rv strain (detected as Rho-H37Rv) by macrophages (detected as FITC-F4/80) was analyzed using flow cytometry. The results shown are representative of three independent experiments. (C) The binding of ficolin-2 with the H37Rv strain leads to the activation of the lectin complement pathway. GST-ficolin-2 or GST proteins were incubated with Mtb H37Rv in the presence of human serum and C4 protein, and C4c deposits on the bacteria were measured as described in the Materials and Methods. The data shown are the mean ± SEM of three independent experiments GST-ficolin-2 group vs. GST group and control group, *p < 0.05. The data were analyzed by ANOVA method.

A previous report indicated that lysophospholipids of Mtb H37Rv may control mycobacterial infection in A549 type II alveolar epithelial cells [30]. We therefore analyzed whether the binding of ficolin-2 to the surface glycolipid portion of Mtb H37Rv could competitively block the adhesion/invasion and infection of Mtb H37Rv in human lung-derived A549 type II alveolar epithelial cells. Flow cytometry (Figure 3A) revealed that the adhesion/invasion of Mtb H37Rv in A549 cells significantly decreased in the presence of ficolin-2 or ficolin A (Figure 3A). These data suggest that both ficolin-2 and ficolin A have significant inhibitory effects on H37Rv adhesion/invasion in A549 cells. Furthermore, we found that the inhibitory effects of ficolin-2 and ficolin A on Mtb H37Rv infection of A549 cells could be reversed by the addition of ManLAM (Figure 3A). This blocking effect occurred in a ManLAM dose-dependent manner (Figure 3A).

### Ficolin-2 can stimulate opsonization of macrophages

Both ficolins and MBL also show opsonic activity via binding of their collagen-like domain to macrophages [13]. To estimate the influence of ficolin-2 on the opsonophagocytic process, rhodamine B-labeled Mtb H37Rv was used as a model bacterium. Macrophages from mouse abdominal cavities were used as phagocytes. Opsonophagocytosis was performed in the presence of recombinant GST-ficolin-2, GST-ficolin-2 plus cytochalasin B (CytB) (a phagotrophic inhibitor), or GST protein. The double-positive cells (FITC-F4/80 and rhodamine B [Rho]), which represented opsonization, were analyzed using flow cytometry. We found that ficolin-2 could promote 17.5% (GST) to 29.2% (GST-ficolin-2) opsonization of the bacteria (Figure 3B). This opsonization represents uptake of the H37Rv strain (detected as Rho-H37Rv) by macrophages (detected as FITC-F4/80). This ficolin-2-induced promotion was inhibited (19.1%) (Figure 3B) by CytB, which is a well-known disruptor of actin polymerization that blocks phagocytosis but not attachment [25]. Our results suggest that the addition of exogenous ficolin-2 significantly enhances macrophage opsonization of H37Rv (Figure 3B), primarily due to phagocytosis.

In addition, in the presence of serum, the highest levels of C4c deposition were observed after mixing ficolin-2 with the H37Rv strain. The complement activation level in the GST-ficolin-2 plus H37Rv group was much higher than that in the control GST plus H37Rv group or the H37Rv group (* *p* < 0.05, Figure 3C). Activation of the lectin pathway occurred in a ficolin-2 dose-dependent manner (Figure 3C).

### Ficolin-2 protects wild-type mice and FCNA KO mice from infection with virulent Mtb H37Rv

To determine whether ficolin-2 has a protective effect against Mtb H37Rv, we examined the anti-TB efficacy of ficolin-2 *in vivo*. Human ficolin-2 cDNA encodes a protein of 313 amino acids with a calculated MW of 35 kDa. DNA segments containing genes encoding the wild-type ficolin-2 protein were cloned in-frame into the eukaryotic expression vector pcDNA3.1 (-) Myc-His, yielding the plasmid pcDNA3.1-ficolin-2. The presence of a 25-residue N-terminal signal peptide sequence indicates that ficolin-2 is a secreted protein.

**Figure 4:**
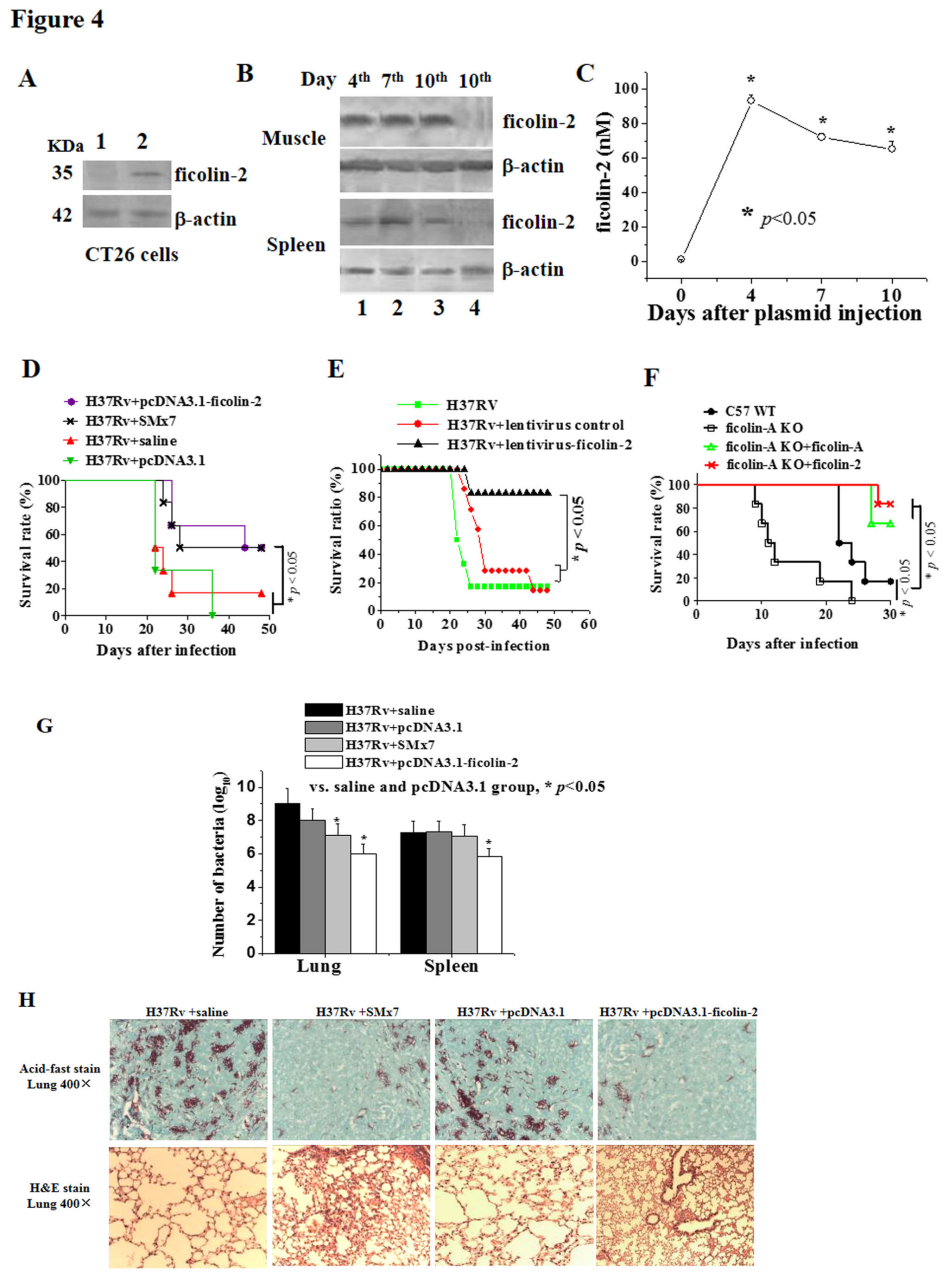
Ficolin-2 prolongs the survival time of both WT and FCNA KO mice infected with Mtb H37Rv. (A) pcDNA3.1-ficolin-2 or empty vector pcDNA3.1 plasmids were transfected into murine carcinogen-induced colon tumor cells (CT26) (H-2^d^), and the expression of ficolin-2 protein in the supernatants at 72 h post-transfection was detected by western blotting with anti-ficolin-2 mAb GN5. β-actin, which is a housekeeping gene with constant expression, was used as an internal control. Lane 1: pcDNA3.1; Lane 2: pcDNA3.1-ficolin-2. (B) Mice were transformed with the pcDNA3.1 empty vector or the pcDNA3.1-ficolin-2 vector (10 µg DNA/mouse) through intramuscular electroporation, and ficolin-2 protein expression in the muscle and spleen tissues were detected on post-injection days 4, 7, and 10 by western blot analysis. Lanes 1, 2, 3: pcDNA3.1-ficolin-2; lane 4 (10^th^ day): pcDNA3.1 empty vector. (C) Ficolin-2 concentrations in sera obtained from mice injected with pcDNA3.1-ficolin-2 (day 0 group vs. other groups, *p < 0.05). Data were analyzed by one-way ANOVA. (D) C57BL/6J mice were challenged with 1×10^6^ CFU Mtb H37Rv per mouse, which was administered on the same day as the vehicles (NS and pcDNA3.1) and pcDNA3.1-ficolin-2. Each group contained eight mice. The survival times of H37Rv-infected mice after administration of pcDNA3.1-ficolin-2 or empty vector through i.m. electroporation on the day of intravenous challenge with virulent Mtb H37Rv were determined. The data shown are the means of five independent experiments with eight mice per group. Ficolin-2-treated group and SM-treated group vs. vehicle (saline or pcDNA3.1)-treated group, * *p* < 0.05. (E) The survival time of H37Rv-infected BALB/C mice challenged with virulent Mtb H37Rv after administration of lentivirus-ficolin-2. Lentivirus-ficolin-2-treated group vs. lentivirus (mock) control-treated and vehicle (saline)-treated groups, * *p* < 0.05. (F) The survival times of H37Rv-infected FCNA KO mice after administration of pcDNA3.1-ficolin-2, pVAX-1-ficolin A, or empty vector through i.m. electroporation on the day of intravenous challenge with virulent Mtb H37Rv. The ficolin-2-treated group and ficolin A-treated group vs. vehicle (saline or pcDNA3.1)-treated group, * *p* < 0.05. The Kaplan-Meier method was used to plot survival curves for each group. (G) Spleens and lungs of Mtb H37Rv-infected C57BL/6 mice were harvested at post-infection day 30, and Mtb H37Rv bacteria were enumerated. The ficolin-2-treated group or SM-treated group (in lung) vs. vehicle (saline or pcDNA3.1)-treated groups, * *p* < 0.05. Data were analyzed by one-way ANOVA. (H) Lung tissues of Mtb H37Rv-infected C57BL/6 mice were harvested at 4 weeks post-infection. The number of Mtb H37Rv bacteria and lung pathological changes were analyzed with Ziehi-Neelsen acid-fast bacillus staining and H&E staining, respectively.

pcDNA3.1-ficolin-2 or the empty vector was transfected into mouse mammalian colon CT26 cells or administered into mice *via* intramuscular electroporation, respectively. Western blot analysis showed that ficolin-2 was expressed in the supernatants of CT26 cells after 72 h of transfection (Figure 4A) and in the extracts of mouse muscle and spleen tissues on days 4, 7, and 10 after electroporation of the plasmids (Figure 4B). The protein was expressed in both tissues and cells as a polypeptide chain with a MW of ca. 35 kDa, which is consistent with the predicted size. The housekeeping gene β-actin was expressed at a constant level. There was no detectable expression of ficolin-2 in the cells or tissues when the cells and mice were transformed only with the pcDNA3.1 empty vector. Ficolin-2 was also detected in serum starting at 4 days after the ficolin-2 plasmid injection (Figure 4C). These data suggested that injection of the ficolin-2 plasmid induced ficolin-2 expression and that ficolin-2 was delivered into the circulation and tissues.

pcDNA3.1-ficolin-2 or empty vector were administered via intramuscular electroporation into C57BL/6J mice challenged with a lethal dose of the virulent Mtb H37Rv strain. The results showed that the pcDNA3.1-ficolin-2 experimental group had a significantly longer (*p < 0.05) survival time compared with the control (saline) group (only H37Rv) and the pcDNA3.1 empty vector group (Figure 4D). The time to 50% mortality (T50) was reached at more than 43 days for the ficolin-2-treated group and the group given seven successive injections (once per day) of streptomycin (SM), which is an antibiotic drug commonly used to treat tuberculosis. However, the T50 was reached at 21 days for the control (saline or pcDNA3.1 empty vector) groups (Figure 4D). Four weeks after infection with H37Rv, the mice were sacrificed to assess bacterial growth. We observed that the viable bacterial counts were lower in the pcDNA3.1-ficolin-2-treated group compared with the control (saline or pcDNA3.1 empty vector) groups, with a statistically significant difference (*p < 0.05) noted in both the lung and spleen tissues (Figure 4G).

Lentivirus-ficolin-2 or pcDNA3.1-ficolin-2 was administered intravenously or *via* intramuscular electroporation, respectively, into BALB/C mice challenged with a lethal dose of virulent Mtb H37Rv. The results showed that the group treated with lentivirus-ficolin-2 had a significantly longer survival time compared with the control group (*p < 0.05, Figure 4E). We observed similar results in BALB/C infected mice treated with pcDNA3.1-ficolin-2 (data not shown).

We also found that the survival time of infected mice was significantly decreased in ficolin A KO C57BL/6 mice compared with wild-type C57BL/6J mice (Figure 4F), and infected ficolin A KO mice in both the ficolin-2 and ficolin A injection groups exhibited significantly prolonged survival times (T50) compared with mice in the un-injected ficolin A KO groups (Figure 4F).

Lung tissues from ficolin-2-treated mice showed significant differences in visible bacterial growth and pulmonary alveoli structure compared with tissues from empty vector pcDNA3.1- and saline-treated mice (Figure 4G and 4H).

Compared with the empty vector pcDNA3.1- and saline-treated mice, acid-fast staining of consecutive lung sections revealed significantly less Mtb bacilli in ficolin-2- or streptomycin (SM)-treated mice (Figure 4H). The lungs in the saline and empty vector pcDNA3.1 control mice showed much more Mtb bacilli with tufted distribution (Figure 4H). In mice, the protective efficacy of one 10-µg injection of the ficolin-2 plasmid was similar to that of seven successive injections of streptomycin (200 µg once per day). H&E analysis revealed that the pulmonary alveolar tissue also appeared to be more intact and contain fewer fused vacuoles in ficolin-2- and SM-treated mice (Figure 4H). The tissue sections in the saline and empty vector pcDNA3.1 control mice showed deterioration, infiltrative necrosis surrounded by mononuclear cells, lymphocyte infiltration, thickening of the alveolar septa, and a severely damaged alveolar structure with missing regions (Figure 4H) based on H&E staining, whereas the ficolin-2 and streptomycin treatment groups showed mild swelling and thickening of the alveolar wall and infiltration of a large number of macrophages and lymphocytes (Figure 4H). Similar data were obtained with BALB/C infected mice (data not shown).

### Ficolin-2 significantly stimulated IFN-γ, IL-17A, TNF-α, IL-6, and NO production by macrophages in vitro

**Figure 5:**
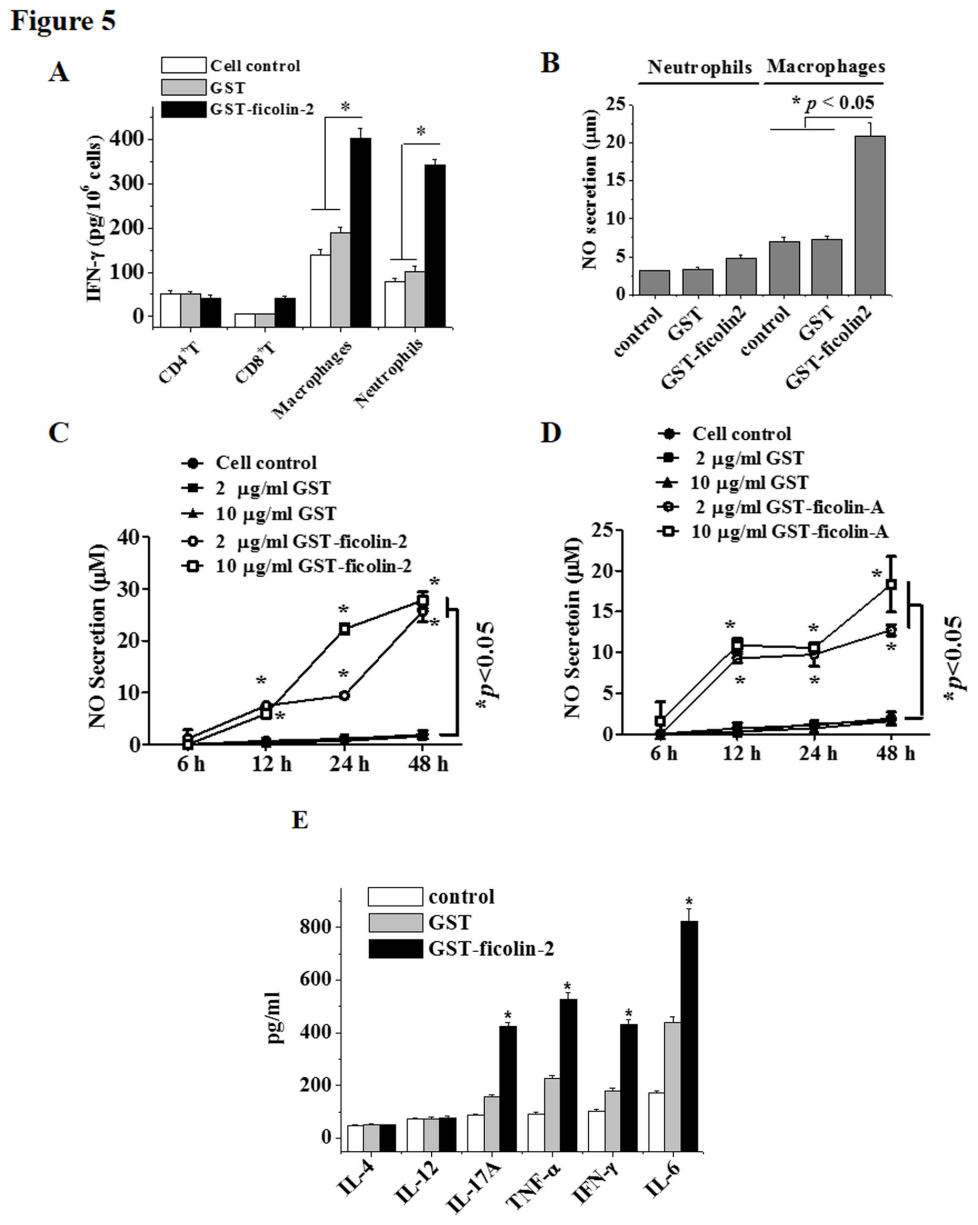
Ficolin-2 significantly stimulated macrophages to produce IFN-γ, IL-17A, TNF-α, IL-6, and NO *in vitro*. (A) For *in vitro* analysis, 1×10^6^ isolated macrophages, neutrophils, CD8^+^ T, and CD4^+^ T cells from untreated BALB/c mice were incubated with 20 µg/ml of GST-ficolin-2 or GST at 37°C for 48 h. The cells were collected and lysed, and the supernatants were collected for analysis of IFN-γ production using cytokine-specific ELISA methods. For neutrophils and macrophages, GST-ficolin-2 group vs. GST group or control group, * *p* < 0.05. (B) Isolated macrophages or neutrophils were cultured in 96-well plates (1×10^4^ cells/well) and stimulated with GST-ficolin2 or GST at final concentration of 20 µg/ml for 48 h. The supernatants were harvested, and the concentrations of NO were measured by ELISA. For macrophages, GST-ficolin-2 group vs. GST group or control group, * *p* < 0.05. (C, D) The indicated concentrations of GST-ficolin2 (C), GST-ficolin-A (D), or GST proteins were incubated with the isolated macrophages (1×10^4^ cells/well) at 37°C for 0, 6, 12, 24, or 48 h, respectively. The supernatants were harvested, and the concentration of NO was measured by ELISA. GST-ficolin-2 group, GST-ficolinA group vs. GST group or control group, * *p* < 0.05. (E) A total of 1×10^6^ macrophages isolated from untreated BALB/c mice were incubated with 20 µg/ml of GST-ficolin-2 or GST at 37°C for 48 h. IL-4, IL-12, IL-17A, TNF-α, IFN-γ, and IL-6 production in the supernatants was measured by cytokine-specific ELISA methods. The data shown are the means ± SEM of at least four independent experiments. All data were analyzed by one-way ANOVA.

We further examined the effects of recombinant ficolin-2 protein on the IFN-γ secretion of purified peritoneal macrophages, neutrophils, splenic CD8^+^ T cells, and CD4^+^ T cells from mice *in vitro*. Compared with GST-treated or untreated control groups, GST-ficolin-2 significantly stimulated IFN-γ secretion from purified macrophages and neutrophils but not from CD4^+^ T cells (Figure 5A). IFN-γ secretion was mildly increased in ficolin-2-stimulated CD8^+^ T cells (Figure 5A). Our data suggest that ficolin-2 directly affects innate immune cells, which primarily include macrophages and neutrophils; however, it had little direct effect on CD8^+^ T cells and no direct effect on CD4^+^ T cells.

Because NO is considered to be an important factor in the innate defense against intracellular pathogens [26], we determined whether ficolin-2 could stimulate NO release *in vitro*. We found that ficolin-2 could significantly stimulate the NO production of macrophages; however, it caused only a minor increase in NO production by neutrophils (Figure 5B). Thus, macrophages were used in the following experiments. We further demonstrated that both ficolin-2 and ficolin A could increase NO release from mouse macrophages over a different time course in a dose-dependent manner (Figure 5C and 5D). These data suggest that ficolin-2 or ficolin A can induce NO release primarily from macrophages, which may lead to enhanced antibacterial activity in the infected cells.

We next further screened the cytokine expression of macrophages after stimulation with ficolin-2. The results shown in Figure 5E reveal that the secretion of IL-17A, TNF-α, IL-6, and IFN-γ was significantly increased after stimulation with recombinant GST-ficolin-2 for 48 h compared with the GST-treated group and the control group, whereas macrophage secretion of IL-4 and IL-12 was not significantly changed.

### Ficolin-2 significantly stimulates proinflammatory cytokine production by macrophages via the activation of JNK

**Figure 6:**
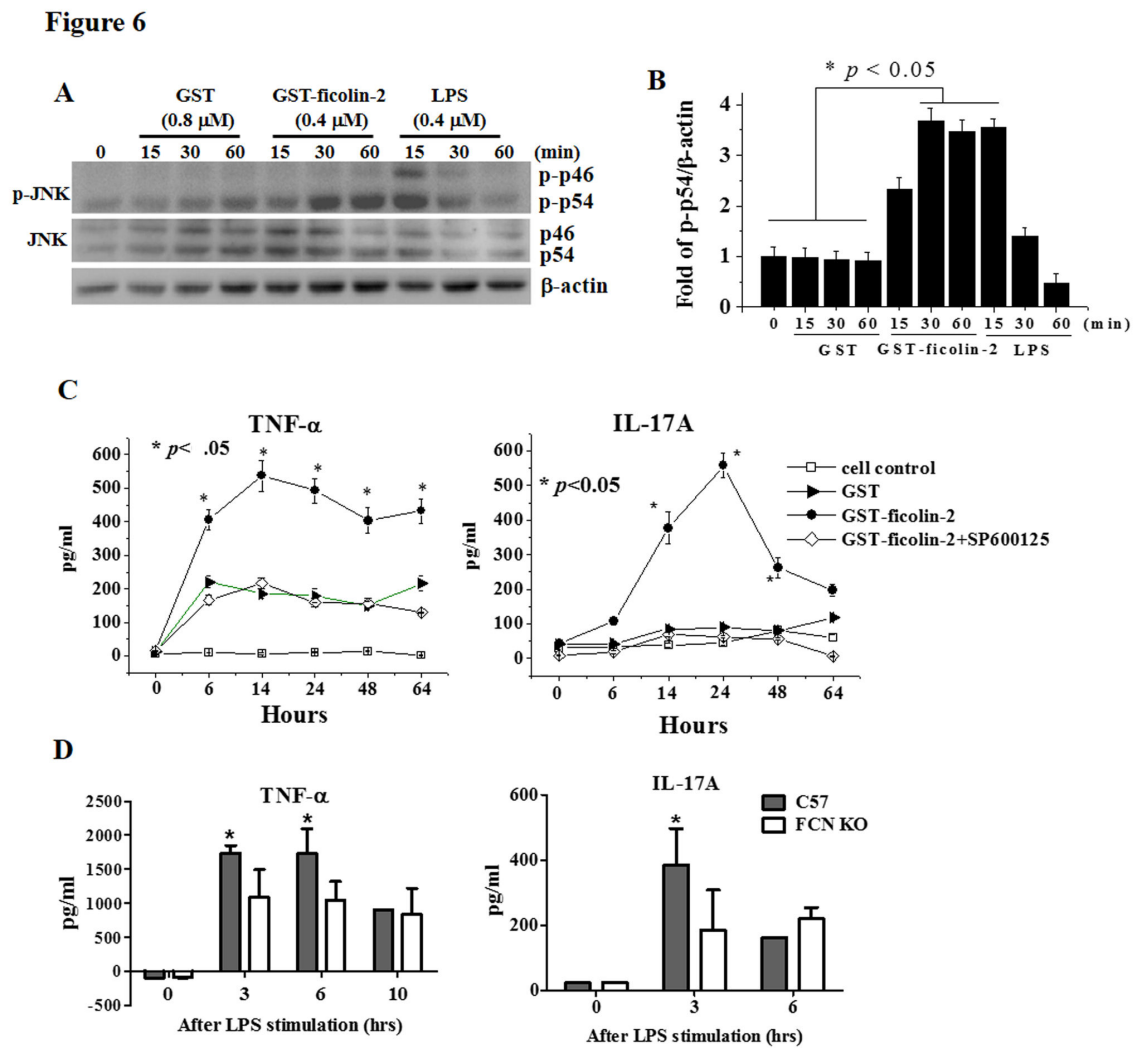
Ficolin-2 stimulated JNK phosphorylation. (A) Murine macrophages (2×10^6^) were stimulated with 20 µg/ml of GST (0.8 µM), GST-ficolin-2 (0.4 µM) or LPS (0.4 µM) at 37°C for 0, 15, 30, or 60 min, respectively. The cells were collected and lysed, and the phosphorylated-JNK (p-JNK) (46/54 kDa) and total JNK were detected by western blotting using the corresponding mAbs. (B) Densities of p-JNK protein shown in (A) were normalized based on the density of the house keeping protein β-actin. The data shown are the means ± SEM of three independent experiments. The data were analyzed by ANOVA. (C) Murine macrophages were cultured in 6-well plates (2×10^6^ cells/well) and subsequently stimulated with 20 µg/ml of GST, GST-ficolin-2, or GST-ficolin-2 plus JNK inhibitor (SP600125, 40 µM, Sigma) at 37°C for 0, 6, 14, 24, 48, or 64 h, respectively. The supernatants were collected, and the concentrations of TNF-α and IL-17A were detected by ELISA. GST-ficolin-2 group vs. GST-ficolin-2 plus SP600125 group, GST group or cell alone control group, * *p* < 0.05. The data were analyzed by one-way ANOVA. (D) *In vivo* analysis of LPS-stimulated TNF-α and IL-17A production. Wild-type C57BL/6 mice (n=8) or FCNA KO C57BL/6 mice (n=8) were injected with LPS (0.15 mg/mouse) via the tail vain. The blood sample (100 µl) was collected from the orbital vein of each mouse at 0, 3, 6 or 10 h. Subsequently, the serum TNF-α and IL-17 levels were examined by ELISA. The data shown are means ± SEM of at least three independent experiments. C57BL/6 group vs. FCNA KO C57BL/6 group, * *p* < 0.05. The data were analyzed by Student’s *t* test.

The above data illustrate that ficolin-2 primarily stimulated macrophages in a direct way, producing the proinflammatory cytokines IFN-γ, IL-17A, IL-6, and TNF-α. We therefore further determined the role of ficolin-2 in the activation of signaling pathways in macrophages. The western blot data showed that the expression of phospho-JNK (p-p54) in mouse macrophages was significantly increased by stimulation with GST-ficolin-2 protein for 30 min or 60 min or by LPS control for 15 min when compared with GST stimulation (Figure 6A and 6B). However, the expression level of total JNK in the samples treated with GST-ficolin-2 was not significantly altered compared with that in GST-treated samples based on western blot analysis (Figure 6A). These data indicate that ficolin-2 stimulates the activation of phospho-JNK in macrophages.

We next determined whether a JNK inhibitor (SP600125) could affect the expression of the proinflammatory cytokines TNF-α and IL-17A, which are stimulated by ficolin-2. In a time course experiment, the expression levels of TNF-α and IL-17A were significantly increased after ficolin-2 stimulation for 6 h based on ELISA (Figure 6C). The IL-17A concentration reached a peak at 24 h, which was later than the time at which p-JNK was significantly activated (30 min; Figure 6A). More importantly, the expression levels of TNF-α and IL-17A were significantly decreased by JNK inhibitor (SP600125) treatment at each time point compared with the controls (Figure 6C). These data indicate that proinflammatory cytokine production in macrophages occurs downstream of p-JNK after ficolin-2 stimulation.

We further examined the production of the proinflammatory cytokines TNF-α and IL-17A in FCNA KO mice. Compared with wild-type C57BL/6 mice, the serum TNF-α and IL-17A levels were significantly decreased in FCNA KO mice at 3 h (for TNF-α and IL-17A) or 6 h (for TNF-α) after treatment with LPS (Figure 6D). However, serum TNF-α and IL-17A were not significantly altered after 6 h (for IL-17A) or 10 h (for TNF-α) in FCNA KO mice compared with wild-type mice (Figure 6D). These *in vivo* data confirm that proinflammatory cytokine production was decreased in ficolin A knockout mice.

## Discussion

Ficolin-2 is an important lectin molecule in serum, and its reduction may contribute to the progression of tuberculosis. In the present study, we found that the serum levels of ficolin-2 were significantly decreased in TB patients compared with healthy controls (Figure 1). The association between ficolin-2 and tuberculosis as revealed by our results corroborates previous data that ficolin-2 insufficiency is associated with recurrent respiratory infections in children [31]. Large ethnic differences in the FCN genes may affect the concentration, structure, and function of ficolin molecules [32]. One study also showed that polymorphisms in the FCN2 promoter were associated with marked changes in the ficolin-2 serum concentration [33]. Further research is needed to determine the relationship between genetic background and concentrations of ficolin-2 in the sera of Mtb-infected patients.

The combination of the fibrinogen (FBG)-like domain and the collagen-like region of ficolin-2 forms a basic subunit consisting of a triple helical tail and a trio of globular heads. The triplet subunits then associate to form higher multimers. The major form in plasma is believed to be a tetramer of subunits (12-mer) [34]. In this study, the GST-ficolin-2 protein was expressed in *E. coli*, and we found that the molecular weight of this protein was more than 300 kDa. This finding indicated that the recombinant ficolin-2 protein was properly folded (Figure 2B). We then found that the recombinant ficolin-2 could bind to Mtb H37Rv (Figure 2C and 2D), and this binding could be blocked by ManLAM, which is a glycolipid of Mtb H37Rv (Figure 2C). Ficolins bind to specific pathogen-associated molecular patterns (PAMPs) on microorganism surfaces and trigger the innate immune response; thus, ManLAM may be the target ligand of ficolin-2.

Next, we confirmed that ficolin-2/ficolin A can significantly block Mtb H37Rv infection of a human lung alveolar epithelial cell line (A549) *in vitro*, which might contribute to host resistance against virulent Mtb H37Rv infection (Figure 3A). ManLAM was able to block the effects of ficolin-2/ficolin A (Figure 3A). We hypothesize that ficolin-2, similar to other lectins such as MBL and SP–D, may inhibit Mtb H37Rv entry by binding to the surface glycolipid ManLAM. Additionally, ficolin-2 may not only initiate the lectin pathway of complement activation but may also perform other biological activities, such as preventing Mtb from targeting lung alveolar epithelial cells. Phagocytosis is a critical step in the Mtb-phagocyte interaction. Ficolins might interact with the macrophage collectin receptor CRT through the collagen-like domains [35]. We also found that ficolin-2 enhanced macrophage opsonization with H37Rv (Figure 3B), which was primarily due to uptake because the process was inhibited by cytochalasin B. We speculated that ficolin-2 inhibited Mtb H37Rv infection of A549 alveolar epithelial cells and stimulated opsonization of macrophages, perhaps through the two different domains of ficolin-2 (i.e., the FBG domain and the collagen-like domain). Ficolin-2 inhibited H37Rv adhesion/invasion in A549 cells via the binding of its FBG domain to the surface glycolipid ligand of Mtb H37Rv, and ficolin-2 also stimulated the opsonization of macrophages, possibly via the binding of its collagen-like domain to macrophages. During opsonophagocytosis, macrophages can kill or limit the replication of intracellular bacteria via lysosomal enzymes and the production of reactive oxygen intermediates (ROI), reactive nitrogen intermediates (RNI), and antimicrobial peptides [36].

Macrophages may undergo classical M1 activation (stimulated by TLR ligands and IFN-γ) or alternative M2 activation (stimulated by IL-4/IL-13) [37]. M1 macrophages are characterized by a high capacity to present antigen, high IL-12 and TNF production and consequent activation of a polarized type I response, and a high level of NO and reactive oxygen intermediate (ROI) production, whereas M2 macrophages typically produce IL-10. Our data showed that ficolin-2 stimulated macrophages to produce high levels of IFN-γ, TNF-α, and NO, indicating that ficolin-2 might activate M1 macrophages (characteristic of Th1-type immunity), therefore stimulating cell-mediated responses that play major roles in the defense against intracellular pathogens [38]. Robinson et al. [39] showed that IFN-γ, TNF-α, and interleukin (IL)-18 cooperated to control the growth of Mtb in human macrophages. Several recent reports demonstrated that IL-17A increased protection against Mtb infection [40]. IL-6 is a proinﬂammatory cytokine, and it is induced by microbial components to support effector T cell proliferation *in vivo* by suppressing regulatory T cells [41]. NO was considered to be an important factor in the innate defense against intracellular pathogens. In this study, we demonstrated that ficolin-2 stimulated IFN-γ, IL-17A, TNF-α, IL-6, and NO production primarily in M1 macrophages, which might contribute to the control of TB infection *in vivo* (Figure 5).

JNK signaling is known to be involved in a wide range of physiological functions, including inflammatory and proliferative responses induced by infection, pro-inflammatory cytokines, and cellular stress [42,43]. We found that the effects of ficolin-2 may depend on the activation of macrophages by JNK (Figure 6) because ficolin-2 activated the expression of phosphorylated JNK and a JNK antagonist (SP600125) decreased the production of IL-17A and TNF-α.

Our *in vivo* experiments revealed that electroporation of a ficolin-2 eukaryotic expression plasmid induced ficolin-2 protein expression *in vivo* (Figure 4A and 4B), and the ficolin-2 protein was delivered into the circulation as well as the muscle and spleen tissues of the injected mice (Figure 4B and 4C). We demonstrated that the administration of exogenous ficolin-2 significantly prolonged the survival time of both BALB/C and C57BL/6J mice infected with the virulent H37Rv strain (Figure 4D–4F) and caused a significant decrease in viable bacterial counts in the lung and spleen tissues of the infected mouse models based on bacterial counting (Figure 4G) and acid-fast staining (Figure 4H).

In addition, ficolin A KO mice had a lower survival rate than wild-type C57BL/6J mice (Figure 4F), and both ficolin-2 and ficolin A injections significantly prolonged the survival time of the infected ficolin A KO mice. To the best of our knowledge, this is the first report demonstrating that ficolin-2 enhances protection against Mtb H37Rv infection both *in vitro* and *in vivo*.

**Figure 7:**
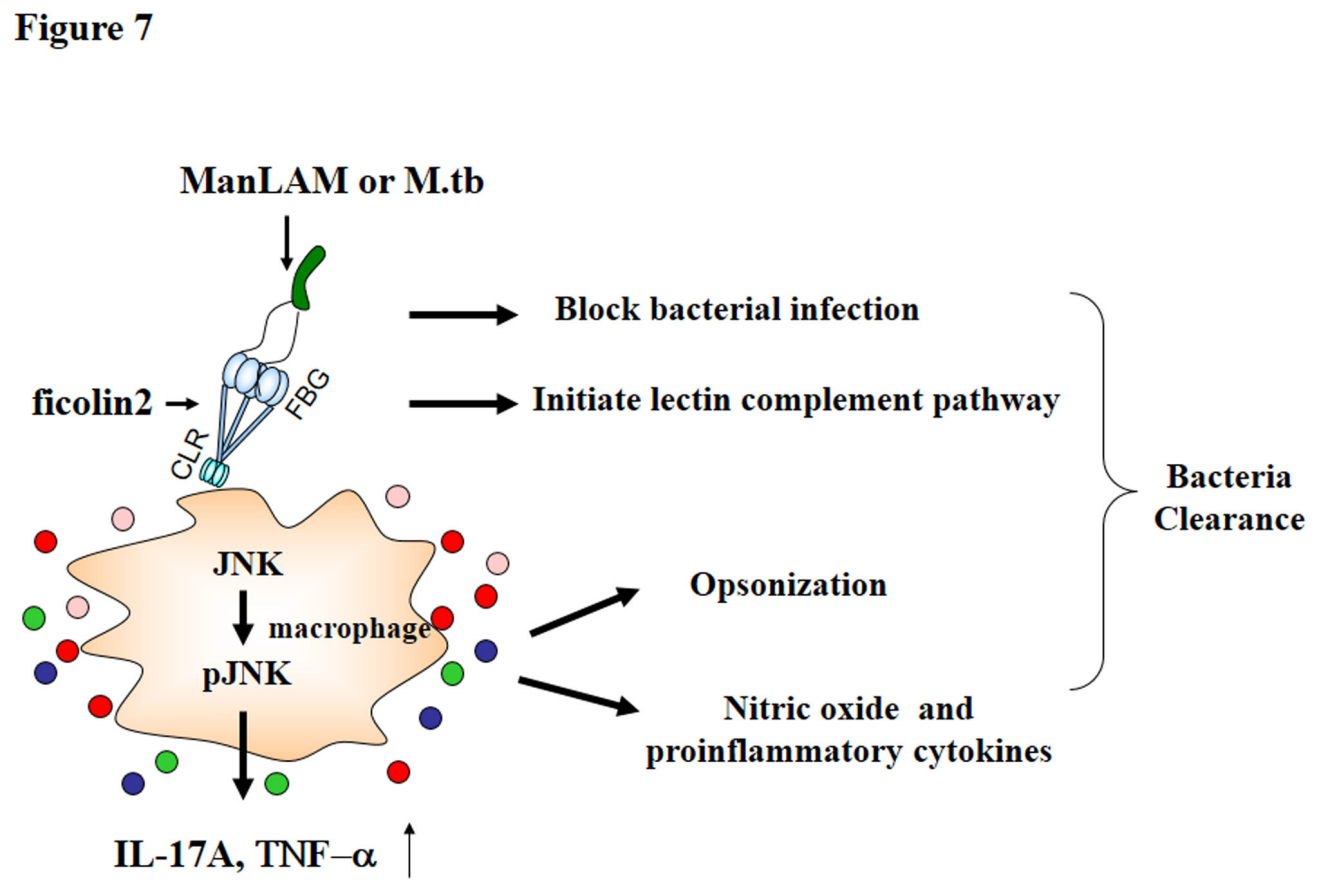
Hypothetical model of the mechanisms mediated by ficolin-2 during Mtb infection.

Previous clinical studies have demonstrated a marked correlation between low serum levels of MBL and immune opsonic deficiency [44]. Our present findings showed that TB patients exhibited significantly lower serum ficolin-2 concentrations, which also suggested that a ficolin-2-deficient host might be more susceptible to infection by these intracellular pathogens. Taken together, our data have demonstrated the potential mechanisms by which ficolin-2 protects against Mtb H37Rv infection *in vivo* (Figure 7). These new findings will contribute to the development of ficolins as novel immunotherapy agents for the prevention of infections or co-infections by these important pathogens.
